# Survival analysis of dental implants placed in a private practice. A multicenter prospective cohort study

**DOI:** 10.4317/jced.61457

**Published:** 2024-05-01

**Authors:** Frederick Li, Marija Roguljić, Ivana Medvedec-Mikić, Mª Ángeles Sánchez-Garcés, Jorge Toledano-Serrabona, Octavi Camps-Font

**Affiliations:** 1 Private Dental Clinic, Vancouver, Canada; 2 Private Dental Clinic, Split, Croatia, Department of Periodontology, University of Split School of Medicine; 3 Department of Restorative Dental Medicine and Endodontics, University of Split School of Medicine; 4 School of Medicine and Health Sciences, Universitat de Barcelona, Barcelona, Spain. IDIBELL (Bellvitge Biomedical Research Institute), Barcelona, Spain

## Abstract

**Background:**

This prospective cohort study aimed to assess the predictability and survival rates of dental implant treatment in edentulous patients while identifying potential factors contributing to implant failure.

**Material and Methods:**

A total of 80 outpatients, receiving 166 dental implants between September 2015 and November 2017 in two private dental clinics, were included in this study. Patient and implant characteristics, surgical procedures, primary stability, prosthetic rehabilitation, failure analysis, and survival rates were analyzed.

**Results:**

The majority of patients (53.75%) received a single implant for treating single-gap edentulism, with 6.25% requiring implants for fully edentulous jaws. Most implants (66.87%) were Avinent Ocean IC implants with specific design features. Surgical placement primarily occurred in healed pristine bone (78.31%), immediate implants in fresh extraction sockets (19.88%), and bone regeneration was simultaneous in 15.66% of cases. While 54.82% of implants achieved primary stability over 35Ncm, none exceeded 45Ncm, and only 4.82% failed to attain primary stability. Prosthetic rehabilitation revealed that 13.25% received immediate loading prostheses. During follow-up, four implants failed, resulting in a 2.41% failure rate, with bruxism (HR: 96.62; *P*< 0.001) and absence of primary stability (HR: 23.54; *P*< 0.001) significantly associated with implant failure. The cumulative survival rate at 24 months was 97.44%.

**Conclusions:**

This study demonstrates the high predictability and survival rates of dental implant treatment in edentulous patients, consistent with established standards. Factors such as bruxism and primary stability may impact early implant failure. Dental implants remain a reliable treatment option, boasting a 97.44% cumulative survival rate at 24 months. Further research is required to explore implant failure indicators and multifactorial influences.

** Key words:**Dental implants, survival, edentulous patients.

## Introduction

The introduction of dental implants in the late 20th century marked a significant advancement in treating edentulous patients (1-4). These implants, capable of osseointegration, offered a groundbreaking solution for patients with missing teeth, leading to the widespread use of implant-supported prostheses. Initially, research focused on optimizing implant surfaces and materials for optimal osseointegration. However, with a better understanding of osseointegration and its high success rate, the focus has shifted to aesthetics and long-term outcomes (5).

Numerous factors, including health conditions (e.g., diabetes, osteoporosis), lifestyle habits (e.g., smoking, alcohol consumption), surgical considerations (e.g., implant placement, bone quality), prosthetic factors (e.g., design, materials), and local conditions (e.g., peri-implant diseases, bruxism), have been suggested to influence implant survival and success (6-12).

Implant survival is defined by the presence of the implant at follow-up, with complications often being biological or, less frequently, mechanical or esthetical. Different studies showed a high overall survival rate, above 95%, after a minimum 10-year follow-up (13). Biological complications encompass issues with supporting tissues, occurring either early or late, with factors like postoperative infections and peri-implantitis contributing to these problems (14). Mechanical complications, though rare (less than 1% of cases), involve device fractures or deformations leading to implant dysfunction, influenced by patient-related factors, prosthetic issues, and implant-related weaknesses (15).

Implant failure can result in functional, aesthetic, and emotional issues, along with financial and time costs. It can also increase infection risk and complicate future dental treatments. To prevent these consequences, regular check-ups, good oral hygiene, and following dental professional advice are crucial. In this study, we aimed to assess the success and survival of dental implants in a private practice while evaluating potential risk factors and conditions affecting implant failure.

## Material and Methods

-Study design

A prospective cohort study comprising a total of 80 outpatients (166 implants), who were consecutively treated between September 2015 and November 2017 in two private dental clinics, was performed. The study design followed The Strengthening the Reporting of Observational Studies in Epidemiology (STROBE) Statement.

-Study population

Patients were given full information about the surgical procedures and treatment alternatives, and informed consent was obtained in all cases. The preoperative analysis included clinical and radiographic examinations (with panoramic radiographs or computed tomography).

All partially or fully edentulous patients seeking implant-supported rehabilitations were assessed for eligibility. The exclusion criteria were general contraindications to implant surgery, such as an American Society of Anesthesiologists (ASA) health status score (20) higher than 3, immunosuppression, bleeding disorders, active treatment of malignancy, drug abuse, psychiatric illness, and intravenous bisphosphonate use. Heavy smokers (i.e., >20 cigarettes/day) and patients under 18 years of age were algo excluded. No restrictions were made regarding the type of edentulism, time of implant placement (immediate, early or delayed implant placement) or loading protocols.

Patients with active periodontal disease received appropriate treatment before the study, following the guidelines of the American Academy of Periodontology (16).

-Intervention

Two experienced clinicians (FL and MR) in dental implantology performed all the surgical and prosthetic procedures.

**Figure 1:**
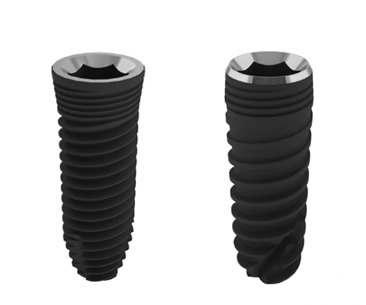
Morphology of used dental implants. A. Avinent Biomimetic Coral. B. Avinent Biomimetic Ocean.

Each patients received at least one commercially available bone-level titanium grade 5 dental implant (Avinent Implant System, Barcelona, Spain) with an internal hexagonal flat-to-flat connection and a sandblasted, and anodized surface (Biomimetic Advanced Surface; Avinent Implant System). Two different macroscopical designs were used: one was a parallel-walled device with a slightly expanded platform and a symmetric progressive thread (Biomimetic Coral, Avinent Implant System, Barcelona, Spain), while the another was a tapered implant with a reverse coronal design and an asymmetric progressive thread (Biomimetic Ocean, Avinent Implant System, Barcelona, Spain) (Fig. 1). The type, as well as the diameter and length of the implants were determined based on the clinician’s criteria according to case requirements.

An antibiotic prophylaxis with 2 g of Amoxicillin was administered to all patients 1 hour prior the surgery, in case of penicillin allergy, 500 mg of azithromycin was given. All surgeries were performed under local anesthesia and followed the manufacturer’s drilling protocols. The choice between one or two-stage implant placement was based on the implant’s primary stability (> 35 N•cm) and the specific characteristics of the case.

In situations where a clinician envisioned an immediate loading protocol, the initiation of obtaining a dental implant impression commenced promptly after the surgical procedure. The placement of prostheses for immediate loading was carried out within a maximum timeframe of 24 hours following the surgery.

The healing timeline was set at 3 months for cases involving pristine bone conditions and extended to 6 months for instances requiring simultaneous bone augmentation. Upon the completion of these intervals, a second-stage surgery was done in cases of implants with cover screw.

The prosthetic workflow encompassed both digital and analog approaches. For partially edentulous patients, zirconia crowns/bridges on a titanium or CrCo base, as well as ceramic crowns/bridges, were performed. In contrast, for completely edentulous patients, acrylic overdentures or acrylic hybrid prostheses were created.

-Variables

During the preoperative visit, demographic data, and medical and dental histories of the patients were collected. Additionally, a periodontal diagnosis was conducted following the classification of the World Workshop on the Classification of Periodontal and Peri-Implant Diseases and Conditions (17).

On the day of surgery, various implant-related variables were recorded: length, diameter, connection size, implant position in the dental arch, implant position in the bone crest (crestal, subcrestal, or supracrestal), insertion torque, and the placement of a closure cap or healing abutment. The need for bone regeneration procedures was also documented. Regarding the prosthesis, information was gathered on the type of impression (digital or analog), prosthetic design (digital or analog), type of restoration (screw-retained, cemented, or cemented-screw-retained), and prosthesis material.

Finally, during the 2-year follow-up, a clinical and radiographic assessment was performed to verify the peri-implant health status. Survival is defined as the presence of the implant in the mouth at the time of examination, regardless of its condition and/or patient satisfaction. On the other hand, failure was diagnosed in case of implant mobility.

-Sample size

Regarding the power analysis, a post-hoc estimation was obtained. A sample size of 166 independent implants provided 96% power at confidence 95% to detect an HR of 4 using a Cox regression model. However, due to the multi-level design of the data (each patient provided at average 2.1 implants), the power was corrected assuming a moderate intra-subject correlation (ρ = 0.5) resulting in a power of 84%.

-Statistical analysis 

Statistical analysis was conducted by a researcher (OC-F) who was not involved in the clinical procedures, using STATA 14 software (StataCorp, College Station, TX, USA).

Subject characteristics were presented as absolute and relative frequencies for categorical outcomes. Normality of scale variables was assessed using the Shapiro-Wilk test, as well as visual analysis through P-P plots and boxplots. In cases where normality was rejected, the interquartile range (IQR) and median were calculated. For variables with a distribution compatible with normality, the mean and standard deviation (SD) were reported.

Cumulative survival rates were computed using the Kaplan-Meier method. Univariate analyses were performed using the log-rank test and univariate Cox proportional-hazards regression for categorical and continuous variables, respectively. These analyses aimed to identify associations between each covariate and implant survival at 24 months. Hazard functions (h) and hazard ratios (HR) with corresponding 95% confidence intervals (95% CI) were calculated for each covariate. The significance level was set at *P*<0.05.

## Results

In the analysis, a total of 80 patients (41 females) and 166 dental implants were included. The mean age of the participants was 58.17 (SD=13.86) years. Details regarding the characteristics of the placed dental implants and the prosthetic restoration are presented in  Table 1 and  Table 2, respectively.

**Figure 2:**
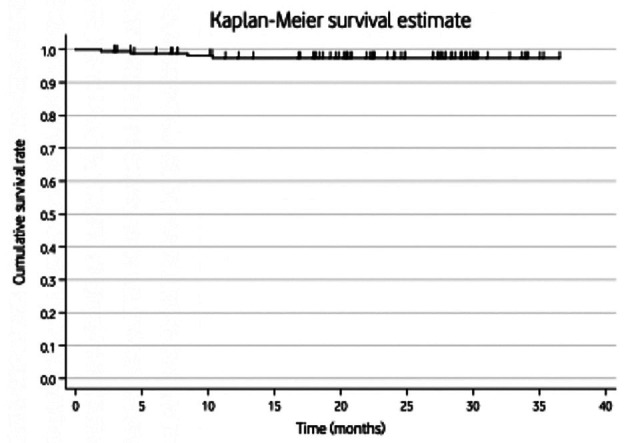
Kaplan-Meier Survival Estimate.

The overall cumulative survival rate was 97.44% (95%CI: 93.30% to 99.03%) at 24 months after dental implant placement (Fig. 2). Regarding failure analysis, a total of 4 implants failed during the follow-up, resulting in a failure rate of 2.41% (95% CI: 0.94 to 6.03). Two implants failed prior to restoration within the first 3 months, while the remaining two failed after the loading of the final restoration. Further details regarding implant failure are summarized in Table 3. Univariate analysis showed a significant association between implant failure and bruxism (HR: 96.62; 95%CI: 9.88 to 945.08; *P* < 0.001) and absence of primary stability (HR: 23.54; 95%CI: 3.28 to 169.16; *P* < 0.001).

## Discussion

This prospective cohort study demonstrated a cumulative survival rate of 97.44% (95% CI: 93.30% to 99.03%) for dental implants used to treat different types of edentulism. Additionally, the absence of primary stability during implant insertion and bruxism appeared to be associated with implant failure. However, our study has several limitations that should be considered. Firstly, a limited sample size and a relatively short follow-up duration were analyzed. A larger sample size and an extended follow-up period are needed to thoroughly assess long-term survival rates and complications of the implants evaluated in this study. Secondly, the collected sample was heterogeneous as it involved different types of edentulism. Nevertheless, the multicentric nature of this study, conducted in private dental practices, enhances its external validity.

Survival, and particularly success, are crucial aspects when measuring the outcomes of implantological treatment. Although numerous publications have proposed criteria for evaluating the success of implant treatment over the last three decades, the parameters suggested by Albrektsson *et al*. in 1986 remain the most widely used within the dental community (18). In our study, the 1-year survival rate of 97.6% reported aligns with rates reported in other prospective studies and meta-analyses (19–21). The first Brånemark’s studies showed a survival rate threshold for implants at 95% in 5 years follow-up. In this line, Wennerberg *et al*. (22) published a systematic review and meta-analysis on 62 observational studies (17,837 implants) with a minimum follow-up of 10 years to assess the risk of long-term failure. The study’s findings revealed an overall weighted survival rate of 96.35% (95% CI: 95.75 to 96.95).

The understanding of implant failure remains a subject of ongoing research, likely arising from multifactorial events hindering implant osseointegration (20). Multiple factors play roles in implant success or failure during preoperative, intraoperative, and postoperative phases. It’s worth noting that the critical period for implant failure occurs within the first 6 months after implant placement, coinciding with the healing period for osseointegration, during which most implant failures occur (20). This temporal aspect is essential to consider when interpreting our results since our study’s follow-up duration is 24 months, encompassing the critical initial 6 months. Unfortunately, we lack data beyond the 12th month when the failure curve should stabilize.

Implant failures can be categorized as early or late, depending on whether they occur before abutment connection or after implant loading, respectively. This differentiation is crucial as these failures have distinct etiologies. Early implant failure results from inadequate bone apposition on the implant, leading to insufficient osseointegration and the formation of soft tissue around the implant. In contrast, late failures are usually associated with peri-implantitis, prosthetic restoration overload, or systemic diseases. In our study, we observed a 50% rate of early failures and a 50% rate of late complications. Although the reasons for these failures remain unclear, they likely result from a combination of multiple factors (23). Bruxism and the lack of primary stability were identified as risk factors for implant failure, aligning with existing literature and systematic reviews on these topics (12,24-26).

Traditionally, it has been proposed that the implant’s insertion torque should be moderate to avoid compromising its osseointegration due to both insufficient and excessive torque. Cobo-Vázquez *et al*. (24) found that only 3.26% of implants placed without primary stability failed, even when the implants exhibited rotation without resistance and lateral oscillation. However, in a recent systematic review (27), it was observed that achieving high primary stability could have a negative impact on bone level stability, although it was not associated with lower implant survival. Consequently, a lack of primary stability may favor micro-movements, while excessively high values can lead to increased bone resorption due to alveolar bone compression. However, except in cases of extraction and/or immediate loading, empirical evidence suggests that achieving stability during implant insertion is relatively less crucial when modified surfaces are used. Therefore, despite the results of our study, it seems that primary stability does not condition implant survival unless an immediate loading protocol is desired (28).

While numerous complications can occur during dental implant treatment, peri-implant diseases are undoubtedly the most significant long-term concerns (29). Peri-implant mucositis and peri-implantitis are highly prevalent complications associated with dental implants, with reported rates of nearly 10% and 30% at the implant and patient levels, respectively (30). However, our study reports lower prevalence rates of peri-implantitis, at 1.8% and 3.75%, likely due to the limited follow-up period. Thus, long-term follow-up studies should evaluate the peri-implant status of these implant in order to know the prevalence of peri-implant disease in a private setting with an specific brand of dental implant (31). In addition, further research is warranted to identify indicator factors for implant failure and explore combinations of different factors or conditions contributing to implant failure.

## Conclusions

Within the limitations of this study, dental implants could be considered a predictable treatment for addressing various types of edentulism, with a survival rate exceeding 95%. However, the habit of bruxism and the lack of primary stability during implant insertion may jeopardize the survival rate of dental implants during the first two years of follow-up.

## Figures and Tables

**Table 1:** Descriptive summary of implants placed.

	N (%)
Nº implants per patient	
1	43 (53.75%)
2	16 (20%)
3	8 (10%)
4	4 (5%)
5	4 (5%)
6	4 (5%)
7	1 (1.25%)
Implant length	
7	5 (3.01%)
8,5	15 (9.04%)
10	67 (40.36%)
11,5	45 (27.11%)
13	32 (19.28%)
15	2 (1.2%)
Implant diameter	
3	1 (0.60%)
3.3	9 (5.42%)
3.5	26 (15.66%)
3.8	30 (18.07%)
4	46 (27.71%)
4.0	11 (6.63%)
4.2	2 (1.20%)
4.5	10 (6.02%)
4.8	2 (1.20%)
5	29 (17.47%)
Arch	
Mandible	60 (63.86%)
Maxilla	106 (36.14%)
Implant position	
Incisor	28 (16.8%)
Canine	16 (9.63%)
Premolar	67 (40.36%)
Molar	55 (33.13%)
Bone density	
I	26 (15.66%)
II – III	130 (78.31%)
IV	10 (6.02%)
Gingival phenotype	
Thin	123 (74.1%)
Thick	43 (25.9%)
Type of bone	
Healed	130 (78.31%)
Fresh socket	33 (19.88%)
Regenerated	3 (1.81%)
Need for bone reconstruction	
No	140 (84.34%)
Simultaneous guided bone regeneration	21 (12.65%)
Sinus lift	5 (3.01%)
Healing	
Submerged	98 (59.04%)
Exposed	68 (40.96%)

**Table 2:** Descriptive summary of prosthetic rehabilitations.

	N (%)
Type of restoration (per implant)	
Bridge	60 (36.14%)
Crown	81 (50%)
Full-arch	13 (7.83%)
Overdenture	8 (4.94%)
Loading protocol	
Delayed	144 (86.75%)
Immediate	22 (13.25%)
Impression	
Conventional	111 (68.52%)
Digital	51 (31.48%)
Protheses materials	
Acrylic	14 (11.11%)
CoCr Base C Crown	45 (27.78%)
Porcelain	7 (4.32%)
Ti Base ZR Crown	90 (55.56%)
Zirconium	6 (3.7%)
Prothesis confection	
CAD/CAM	49 (30.25%)
Conventional	113 (69.75%)

**Table 3:** Failed implants description.

ID	Nº of implants	Age	Gender	Smoking	ASA	Periodontal health	Implant position	Bone density	Implant site	Phenotype	Implant	Diameter	Length	Torque	Primary stability	GBR	Phases	Load	Bruxism
1	4	53.18	F	0	I	Health	24	II-III	healed	thick	Ocean IC	4	11.5	<35	No	no	Sugmerged	Delayed	Yes
2	1	49.65	M	0	I	Health	21	II-III	healed	thick	Ocean IC	4	13	<35	No	no	Sugmerged	Delayed	Yes
3	2	73.71	M	>10	I	Health	12	II-III	post-exo	thick	Ocean IC	4	11.5	35-45	Yes	no	Sugmerged	Delayed	Yes
4	3	61.78	F	>10	I	Health	35	II-III	healed	thin	Coral IC	3.3	11.5	35-45	Yes	no	Exposed	Delayed	No

## Data Availability

The datasets used and/or analyzed during the current study are available from the corresponding author.
